# Refugee’s agency and coping strategies in refugee camps during the coronavirus pandemic: ethnographic perspectives

**DOI:** 10.1186/s40878-022-00302-3

**Published:** 2022-08-25

**Authors:** Claudia Böhme, Anett Schmitz

**Affiliations:** University of Trier, Universitätsring 15, 54296 Trier, Germany

**Keywords:** Refugee camps, Covid-19, Germany, Lesvos, Moria, Kakuma, Kenya, Agency, Coping strategies, Social Media

## Abstract

The global spread of the coronavirus pandemic has particularly dramatic consequences for the lives of migrants and refugees living in already marginalised and restricted conditions, whose ongoing crisis is at risk of being overlooked. But refugees are not only extremely vulnerable and at risk of infection, as several reports show, quickly develop their own protection measures like the production of hygienic products, the publication of their situation and calls for action and help. Therefore, this paper aims to research the effects of the coronavirus crisis on refugees in camp settings with a special ethnographic focus on how refugees actively deal with this crisis and if they, through already developed resilience, are capable of adapting to the restrictions as well as inventing strategies to cope with the difficult situation. To account for the variety of refugee camps as well as the different living conditions due to their locality, history and national asylum politics, we will look at three different locations, namely refugee asylum homes in Germany, hotspots on the Greek islands as well as one refugee camp in Kenya. The main questions will be how, under structurally and institutionally framed conditions of power and victimisation in refugee camps, forms of agency are established, made possible or limited. The goal is to show which strategies refugees apply to cope with the enhanced restrictions and exclusion, how they act to protect themselves and others from the virus and how they present and reflect their situation during the coronavirus pandemic. Finally, this discussion offers a new perspective to consider refugees not only as vulnerable victims, but also as actively engaged individuals.

## Introduction

According to the United Nations High Commissioner for Refugees (UNHCR), at the end of 2019 79.5 million people were forcibly displaced worldwide, of whom 26 million are officially registered as refugees (UNHCR, 2020a). While most of the refugees seek shelter in urban areas or informal dwellings, only 2.6 million live in refugee camps (USA for UNHCR, 2021). Since the 1980s, refugee camps are increasingly in the focus of scientific research and critical reflection. They have been conceptualised as a specific form of the institution “camp”, which in recent global history has represented a special type of the exertion of violence and domination (Greiner & Kramer, 2013). Refugee camps were theorised in the context of social processes of power, control and security (Pieper, 2008) and described as “total institutions” (Goffman, 1961) that normatively function according to their own rules (Foucault, 1977, 1984). As “non-places” (Augé, 2008), they are said to be characterised by a lack of history, relation and identity. But as many studies show, most refugee camps, although originally intended to be temporary, have become long-term dwellings, “accidental cities” (Jansen, 2011, 2018) and city-camps (Agier, 2002) and homes with people living there for an average of 17 years (Devictor, 2019; Feldman, 2015; Herz, 2013). This means they have indeed become places with their own history, relation and identity. As "quasi total institutions" (Schmitz & Schönhuth, 2020), refugee camps are characterised by their structurally and institutionally determined conflict and violent situations, restrictions, regulations and exclusion (Böhme & Schmitt, 2022b).

The global spread of the coronavirus since the beginning of 2020 further exacerbated the difficult situation of migrants and refugees. The closing of national borders and the concentration on one's own “nation” has hindered mobility to safe places and restricted their rights to asylum and durable solutions (Böhme & Schmitz, 2020). During the coronavirus pandemic, the inhabitants of refugee camps are under severe threat as the virus can easily spread under encampment conditions. They are at risk of further social exclusion or violence as refugees are blamed for spreading the virus in local communities.

This paper aims to discuss the effects of the coronavirus crisis on the inhabitants of refugee camps, and the high risk of infection, which leads to further restrictions and limitations to their already difficult living conditions. We will ask how, against the background of the pandemic, under the given structurally and institutionally framed conditions of power and victimisation in refugee camps, forms of agency are established, made possible or limited. The goal is to show how refugees deal with the crisis within a crisis and to discuss if they, through already developed resilience, are capable of adopting and inventing strategies to cope with the difficult situation. Using the theoretical perspective of relational agency (Emirbayer & Mische, 1998), we want to show how actors create the enabling capabilities of social practices through their individual agency (Raithelhuber, 2012: 143) within restrictive structures. This theoretical perspective highlights the social practices of refugees dealing with conflicts and restrictions as a "capacity to act" and "power to act" (Raithelhuber, 2008: 18) of an individual or a group to achieve change through their own decision to act (Raithelhuber, 2012: 130). This discussion offers a new perspective to consider refugees not as either vulnerable or active, but as vulnerable and engaged individuals at the same time. Many scholars (Stronegger, 2020; Zarka, 2020) have stated that the coronavirus pandemic demonstrates a kind of Foucauldian “biopower” and “biopolitics” (Foucault, 1977) through which citizens are not subjects of law, but a biological population to be controlled by means of epidemiological (biostatistical) surveillance (Arminjon & Marion-Veyron, 2021). According to Giorgio Agamben modern policies and practices of the state take control over the bodies of citizens as the ‘biopolitical paradigm of the modern’. In times of emergency, Agamben argues, the modern state resorts to the ‘state of exception’, in which the bare life of citizens is subject to unmediated power (Agamben, 1998:69,99 and Agamben, 2005: 1–3 cited from Van den Berge, 2020:3). This discussion can be made useful against the background of the coronavirus pandemic using the theoretical perspective of agency. With the theoretical frame of relational agency, we aim to show that refugees are able to overcome this biopolitical power as a system of constant surveillance and discipline through different coping strategies, knowledge and practices.

The empirical data of this paper is based on our research in three different refugee camp settings, namely refugee asylum homes in Germany, a “hotspot” on the Greek island of Lesvos as well as a refugee camp in Kenya. This comparative perspective shall account for the variety of refugee camps across the globe as well as the very different living conditions due to their history, institutional structures and national asylum politics.

With our findings and discussion, we aim to contribute to the growing research on migration, refugees and refugee camps with a comparative, international and interdisciplinary approach. This is of great relevance not only for the study of migration and refugees, but also for the public and global society as a whole, in finding long-term solutions for the ongoing migration crisis.

## Methodological approach

The paper is based on field research in Germany, Greece and Kenya between 2018 and 2020. While we started with ethnographic fieldwork on the ground, with the beginning of the coronavirus pandemic, we had to change and adapt our fieldwork to the use of digital ethnography (Kozinets, 2002), research at distance as well as fieldwork in dangerous surroundings and under conditions of crisis. Therefore, our research design is a mix of digital and classic ethnographic-participatory methods. During 14 months of field research in three asylum homes (one initial reception centre and two follow-up reception centres) in Germany between March 2018 and May 2019, the authors conducted research on the causes of conflicts in the facilities and the introduction of a cultural sensible complaint management system for refugees (Böhme & Schmitz, 2022a; Schmitz, 2020a). The field research comprised participant observation in the refugee accommodations, shadowing (Czarniawska, 2007) social workers during their daily work, informal conversations with refugees, as well as formal interviews with administrative managers, social workers and facility administrative staff. With those inhabitants, to whom friend and trust relationships were built-up over time; formal interviews were undertaken regarding their migration biographies and their experiences during their time in Germany. Even before the coronavirus pandemic, we have used digital messenger tools such as WhatsApp and platforms like Facebook to stay in touch with our contacts and follow their migratory journeys after they had to leave the homes of first admittance via transfer to another region or due to deportation. In addition, some residents who had a key role in the homes due to their language skills or personal competences and with whom trust was established had become our "field assistants." The involvement of refugees as field assistants played an important role in our research, which helped us gain access to private rooms and provided the authors, while not in the camp, with important information on conflicts and problems in the institution through WhatsApp text and pictures. Due to increasing restrictions because of the coronavirus pandemic since March 2020, our field research in the German asylum homes was severely restricted: all facilities were closed to visitors and under strict control. For this reason, we resorted to the digital possibilities to conduct research at distance. On the ground fieldwork was supplemented and later on replaced by digital ethnography, which meant communicating with our contacts in the field via smartphone and messenger services, following their social media postings as well as online research on news around the asylum homes and refugee camps.^1^ We received important information about the situation in the camps via e-mail communication with administrative staff and via WhatsApp from refugees. Our contacts among the inhabitants sent us regularly information, pictures and reported about the pandemic situation in the camp and about the conflicts among residents, which had intensified during the lockdown. In June 2020, the authors were allowed to visit one of the facilities, interviewed the management of the facility and carried out short-term observations concerning the visible hygienic measures and the living conditions during the coronavirus pandemic on site.

During a field trip to Kenya and Tanzania in 2017 in preparation for her research project,^2^ Claudia Böhme talked to urban refugees in Mombasa and Nairobi and visited the Kakuma refugee camp. During her visit to the camp, she talked to several inhabitants and since then has been in regular contact with them and other camp inhabitants of their social network via mobile telephony, WhatsApp, Facebook, Instagram and Twitter. Since conducting fieldwork on site became impossible due to travel restrictions, Böhme started digital ethnography on social media platforms with individual inhabitants of Kakuma or camp led organisations.

The coronavirus pandemic especially intensified controls and restrictions of the EU border regime and with it the situation of thousands of refugees stuck in places like on the Greek island of Lesvos. As field trips were not possible due to mobility restrictions, we first started digital research on the situation in Lesvos. Via e-mail and social media like Facebook and Twitter, we established contacts with relevant organisations like the UNHCR and local and international NGOs, to receive information about the situation of the refugees in Moria during the crisis. When Lesvos was no longer classified as a coronavirus risk area, we used this opportunity to do field research on the ground. In September 2020, both authors went on an exploratory field trip to Lesvos, Greece to study the effects of the coronavirus crisis on refugees in camp settings and by coincidence witnessed the burning of the camp Moria on 9 September 2020, as well as the subsequent situation on the island. Just the day before the fire broke out, they talked to NGOs and visited important refugee aid centres in Lesvos, like the Mosaik community centre (lesvosmosaik.org, 2021), met one of the founders of Sports for Refugees.org. and visited the nearby Moria village. After the fire broke out, we visited the area of the destructed camp and the closed street near Kara Tepe, where we talked to refugees who had to camp on the streets, to aid workers and journalists working in the area. We also talked to the local population in the town of Mytilini as well as refugees who were allowed to come to town (de Mos, 2020). Since then, we were in contact with refugee actors and NGOs via Email, Skype, Facebook and WhatsApp and have followed their online reports.

### Refugee’s agency under vulnerable conditions

Among the public, refugees (living in camps) are either viewed as vulnerable victims or cunning crooks (Horst, 2006: 92–103), agency is in this way only discussed with the latter and as a rather negative habit. Within the first narrative, refugees are labelled as passive, vulnerable "objects" that simply need to be administered legally (Wihstutz, 2019: 15). This presents refugees in an overall situation of powerlessness, as if they had no capacity to act anymore and would be completely subject to their desperately powerless and precarious situation. The other picture of the refugee is that of a cheater, who acts untruthfully or illegally to get access to aid and resources (Horst, 2006: 93–98). While there has been a shift in global policies in regard to refugees’ agency from vulnerable to capable actors and to promote their self-reliance and resilience, aid agencies like the UNHCR still produce binary categories of refugees or whole groups of refugees as either vulnerable or self-reliant. In the hierarchical relationship of power between givers and receivers’ refugees are viewed as only “actors to be” through the help of donors (Krause & Schmidt, 2020). As many studies have shown, refugees should no longer be conceptualised through these binary oppositions either as vulnerable or actors, victims or cheaters, but as people who can be vulnerable and active subjects at the same time, who take action and explore the possibilities of their own agency in the most difficult and challenging situations (Fritsche, 2012; Geiger, 2016; Horst, 2006; Hutchinson & Dorsett, 2012; Jansen, 2016a, 2016b; Kibreab, 2004; Omata, 2017; Schmitz & Schönhuth, 2020; Turner, 2015). From this view, refugees have decision-making power and actively shape their lives and futures despite the institutional power relations in the camps (Benz & Schwenken, 2005; Schäfer, 2015). This perspective highlights the concept of relational agency, which suggests that agency is no longer attributed to individual persons, but is produced in social relations and relationships between persons or between persons and objects, in a network of "human" and "non-human" actors (Raithelhuber, 2012). In their analyses of agency, Emirbayer and Mische understand “human agency as a temporally embedded process of social engagement, informed by the past (in its habitual aspect), but also oriented towards the future (as a capacity to imagine alternative possibilities) and towards the present (as a capacity to contextualise past habits and future projects within the contingencies of the moment)” (Emirbayer & Mische, 1998: 963). In this temporal perspective, individuals can take different agency orientations related to different structural contexts. “As actors move within and among these different unfolding contexts, they switch between (or “recompose”) their temporal orientations—as constructed within and by means of those contexts—and thus are capable of changing their relationship to structure” (Emirbayer & Mische, 1998: 964). This ability to act allows that, particularly in crisis or conflict situations, individuals are able to think beyond established routines, develop larger capacities for creative and critical interventions (Emirbayer & Mische, 1998: 1007) and take different positions within the networks (Schmitt, 2019: 283). In using this understanding of agency for the structures in refugee camps, action is not seen as a pure product of structures, but rather as a potential for empowerment of the actors within these power structures (Fritsche, 2012: 368). In this way, refugees are not presented as powerless administrative victims and their actions as the pure product of structures (Schmitz & Schönhuth, 2020) but as individuals who are able to cope with a structurally felt situation of powerlessness.

This also relates to traditional coping strategies, which many refugees have developed during different phases of their migration or over the course of their entire lives in which they have experienced insecurity and violence. Many refugees come from states with a history of war, bad governance or dictatorships characterised by failing to protect their citizens; or from regions where the state itself is the actor of prosecution and discrimination. They also may come from regions with difficult economic conditions with high rates of unemployment and minor chances to be sustainable and/or with challenging climatic conditions—regular droughts or floods, causing hunger and disease. Moreover, their migration histories and life in transit zones or exile often encompass a long period of time of up to several years, centuries or generations (Feldman, 2015; Hanafi, 2008). Horst (2006) for example has shown how Somali refugees in Dadaab cope with their difficult and often insecure lives in the camps. Coming from a culture and heritage of nomads, according to Horst, the Somali possess various social security mechanisms and coping strategies: namely huge solidary transnational social networks for cooperation and support, a culture of mobility in looking for greener pastures as well as travel practices (for religious or trade reasons). A third strategy is the diversification of options, which means that during difficult times they are able to choose from various options based on their daily experience to take the best possible decision (Horst, 2006: 62–73).

This perspective can be especially productive in the context of refugees living in camps in times of the coronavirus pandemic, when coping strategies are highly needed. Our research during the time of the coronavirus pandemic shows the everyday agency and coping practices of refugees living in camps in dealing with institutional, structural pressures and risks of health and social unrest. These strategies were adopted in different ways depending on the context, individual ability and social constellation as well as a practical (functional) knowledge of action and survival strategy.

### The case of Germany´s asylum homes during the coronavirus pandemic

The case of Germany is a representation of the situation in a welfare state in the Global North, which received growing public attention through “the refugee crisis” in 2015. Since then, federal governments had to reinvent and restructure their asylum policies to cope with the high influx of refugees from Syria, the Middle East, Asia and Africa. The political, civil society and academic discussions in Germany have focused on the question of how the asylum and reception system can be improved to adapt to the needs of both refugees and the receiving society (Grote, 2018). The German asylum system often remains strange and incomprehensible (SVR-Forschungsbereich, 2018). However, the importance of system knowledge through information and clarification is particularly important for refugees in order to remain agency capable (SVR-Forschungsbereich, 2018: 6).

In Germany, asylum seekers are first accommodated and registered in so-called (first) reception centres or collective shared accommodation (BAMF, 2020). These forms of accommodation have an ambivalent character: on the one hand, they offer protection for refugees through their services and structures; on the other hand, they represent the optimization of a system of mass rejection and signify “border zones” (Böhme & Schmitz, 2020) for the inhabitants because of their specific exclusion practices. These exclusive mechanisms are characterised by a de facto non-integration of inhabitants into the local society. The refugees have no access to the labour and housing market and only access to health care and education within or under the control of the reception centres (Böhme & Schmitz, 2020).

Life in these accommodations is restricted and regulated, as inhabitants have to share rooms, sanitary and health facilities with a large number of people from diverse social, ethnic and cultural backgrounds as well as different habits. The coronavirus pandemic has led to further restrictions and major health threats, reflected in the early warnings and reports from media, refugee organisations and NGO's. In a 12–14 square meter multi-bed room with three to six other “strangers” (Böhme & Schmitz, 2020), it is impossible to maintain a physical distance or a specified safety distance. The existing communal kitchens and washrooms are used by all residents, which means ideal conditions for the virus to spread. Food distribution and shower facilities are regulated by the accommodation for certain times of the day and are located outside of the living space in hallways or other houses. The lack of important protective measures such as masks and disinfection products, as well as the lack of cultural sensitive, clear and understandable information and explanation about the virus, is leading to fear, panic, protests among the inhabitants of the facilities, conflict and violence as well as threats and prosecution by security staff and police (Riese, 2020).

In April 2020, the media reported on the rising cases of infection occurring in refugee accommodations all over Germany and the increasing numbers of asylum homes that were put under quarantine (Hill & Schmitt, 2020). The quarantine conditions complicated the situation, increasing the feeling of powerlessness and isolation among the inhabitants. Staying in the rooms for 14 days without direct outside contact, the potential for conflicts, violence and active resistance rose due to fear of infection or misbelief of the coronavirus pandemic and mistrust in the preventive measures taken (Riese, 2020). In the following, we will describe how administrators and inhabitants of refugee accommodations in one region reacted to the coronavirus pandemic and to the outbreak of the virus in the respective institutions. Our special focus will be on the first measures taken by the camp administrators, the consequences and reactions of the inhabitants and their agency and coping strategies during the course of the coronavirus pandemic.

### Protective measures in asylum homes in one of Germany’s federal states

As the federal government was rather slow to react to the coronavirus pandemic, the head of the asylum homes had to develop their own hygienic and health protective measures. As one of the first measures, a visiting ban was imposed and travelling to relatives and friends restricted. Refugees who had left the homes as well as newcomers had to go into quarantine for 14 days. Cleaning and disinfection intervals for communal rooms such as toilets and washrooms were increased and hygienic products distributed to the inhabitants. The safety distance of 1.5 m was marked on the floors in public areas and guarded by the staff. The occupancy of the rooms and the overall occupancy of the facilities was reduced from 75 to 50%. The residents were informed about the coronavirus pandemic either through written notices and posters or personally by the staff or hired translators and provided with advice to protect against the virus. Individual building complexes with separate rooms and toilets were prepared for patient care. Vulnerable people who belong to risk groups such as sick, immune-deficient, pregnant women and families with children were transferred to the communities. All refugees with symptoms of the disease were tested. All newcomers were quarantined for 14 days in so-called “separation rooms”. The already limited social life and the few social activities in the asylum homes were further restricted: schools, playgrounds and playrooms, tearooms, women's cafes, gyms and places for social projects closed. As respiratory masks were not provided for the inhabitants at the beginning, refugees had to produce their own masks out of second-hand clothing. In this way, refugees were able to use their potential and competences (e.g. tailoring knowledge) to invent protective measures. Even if there was no requirement to wear masks within the facility, many residents did so. In the next chapter, we would like to show by means of an example, how the inhabitants reacted to the outbreak and to the quarantine and which coping strategies and forms of agency they created under the conditions of increased restrictions, mistrust and fear of infection.

### Mistrust, conspiracy and conflict—refugees’ reaction and coping strategies during the outbreak of the virus

On 25 August, the first corona case was registered in one of the asylum homes where we were doing research. A young inhabitant showing typical symptoms was tested positive for the virus. Shortly after, 43 contact persons of the young man were identified and isolated in a “quarantine house”. The whole asylum home was put under quarantine for 14 days and all refugees were tested. Every inhabitant received a letter (in German!) about the obligatory quarantine measures as well as advice to download and use the German “Corona-Warn-App”. In the following days and weeks, the number of coronavirus cases added up to 65 persons out of 519 inhabitants.

We received the news from David,^3^ a young man from Nigeria who we have known since he came to Germany via Italy in March 2018, but who was denied asylum due to Dublin law. Since then he had been transferred to other accommodations twice and was once deported to Italy but managed to come back. When talking about the pandemic, David was very ambivalent. While he first respected the overall information and measures in Germany, he did not believe that the Coronavirus existed in his home country Nigeria and said that pandemic was used there only as a tool to enforce political measures.

Upon hearing of the first coronavirus case David was extremely worried especially as he was not in the asylum home at that time but at his girlfriend’s place. He didn’t know what to do: to return to the asylum home would mean immediate quarantine for the next 2 weeks, not to return would mean to receive a fine (reduction of pocket money) and later quarantine. Finally, he decided to return to the asylum home, to be tested and quarantined. After some days in quarantine, he called one of the authors because he was so worried and confused by what had happened and, like many others, did not trust the asylum home administration anymore. This mistrust was fostered by several facts: First, inhabitants had received their results of testing quicker than they were told, second, the numbers of infected persons in the news differed from their own observation in the asylum home. David also wondered why people who were tested positive were not receiving any treatment or special care. Further, a man had received a positive test result although he had not been in the facility when people were tested. According to his perception, these mistakes and inconsistencies would not be possible in a strict and correct country like Germany. This was supported by circulating fake news refugees received via social media also from their countries of origin, where trust in government or health organisations is usually low. Inhabitants became extremely suspicious and no longer believed what they were told. “Is it corona at all or only politics?” “Do they get money for this?” David asked.

Apart from those incidents, the quarantine was a time of isolation and unrest. The inhabitants had to stay in their small rooms and only within a certain time slot go to the outside area. Visitors were not allowed in, but could bring food or needed things to the camp entrance. David told us how boring the time was and how happy he was when his girlfriend brought food for him. Only through his mobile phone could he stay connected to his friends in Germany. As people were breaking the house or quarantine rules nearly every day when they tried to smoke or were reluctant to stay inside, conflicts between inhabitants and between inhabitants and staff increased. Moreover, the situation also fostered violence between groups of refugees when mentally unstable refugees became violent against themselves or others.

As David told us, after the 14 days of quarantine was lifted, it was like a party for the inhabitants of the asylum home: the inhabitants celebrated, ran outside and left the asylum home for the weekend as early as possible. The news reported that doctors had officially proven that the inhabitants were all healthy. On-site, the obligation to wear a mask outside the rooms and the visiting ban remained. First in 2021 and due to inhabitants’ complaints, the Wi-Fi network was expanded in the homes and notices on relevant information from the internet was displayed in several languages. To continue with education in the camp, online German language courses via smartphones were offered. Since the second lockdown and according to the new regulations on face masks, surgical masks were distributed to all inhabitants.

The example shows that life in the asylum homes in Germany was severely restricted and subjected to administrative rules. In this case, refugees’ agency played out in different forms of resistance. While at the beginning of the pandemic, refugees would still engage in protective measures like producing masks and protecting themselves, over time many inhabitants mistrusted the management of the homes as their perception of Germany and its strict bureaucracy did not fit to the slow reaction, missing resources, disarray and mistakes made during measures taken. As information was scarce, not well-directed or culturally insensible, refugees turned to and trusted more the information they gained through their social networks and social media. To not lose their control over the situation, they constructed and circulated their own narratives and interpretations of the coronavirus pandemic and narratives of suspicion soon circulated in the homes. The implied measures of visiting bans, quarantine reinforced fear, feelings of powerlessness, and being oppressed. This tension became visible through rising (violent) conflicts between inhabitants. Refugees acted by opposing the measures, like staying away from the homes, escaping or violently protesting against quarantine. In this way refugees used resistance contextually and situationally to actively circumvent restrictive structures under pandemic conditions.

### The coronavirus pandemic in the camps at the European borders: the case of Moria

The second case is the refugee camp Moria located on the Greek island of Lesvos. The camp was the largest refugee camp at the external borders of the European Union (EU) in the Mediterranean, where many refugees were stuck.

Ziegler (2020) considered it as “The shame of Europe” due to the horrible living conditions in the camp, as well as the disregard for human rights and EU asylum law caused by failing camp administration and irregular bureaucracy (Balouziyeh, 2017; Rozakou, 2017; Ziegler, 2020). Conceptualized as a temporary registration and transit centre Moria had been transformed into a EU hotspot and had become an “anti-shelter” caused by the “spectacle of deterrence” (Howden, 2020) which was characterized by a “politically crafted materiality of neglect” (Pallister-Wilkins, 2020). The former military camp had a reception capacity for about 3000 people. However, at the time of our research approximately 13,000 people^4^ were living in the camp and in the so-called “jungle” in the surrounding olive groves. Even before the coronavirus crisis, camp Moria was making headlines and was subject to political and public debates because of the inhuman living conditions in the camp.

In the massively overcrowded camp, maintaining social distance was impossible and the hygienic situation was already worse before the coronavirus crisis due to insufficient water supply, sanitary and medical facilities (Omid, 2020). The coronavirus crisis further complicated this situation: necessary evacuation measures were stopped or fulfilled only to a minimum. The evacuation of children and young people from Lesvos, planned by eight EU states, had been postponed.^5^ At the beginning of the coronavirus pandemic, civil society alliances drew attention to the deterioration of living conditions and warned of an outbreak of the virus in the camp, calling on the EU to act (AG Migration et al., 2020; Europe must Act, 2020; #LeaveNoOneBehind, 2021; Zoch, 2020).

**Fig. 1:**
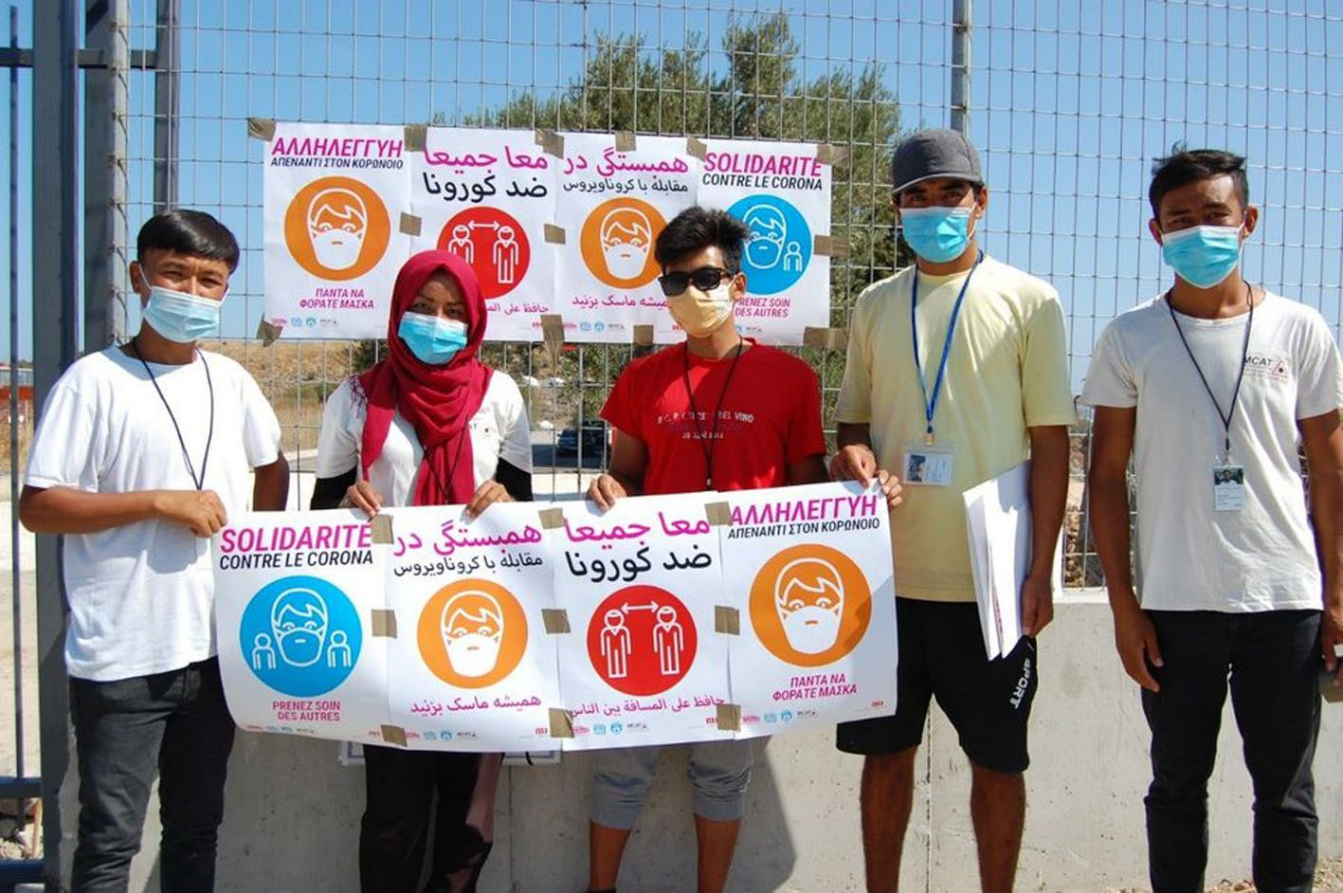
Moria Coroan Awareness Team; Copyright MCAT 2020; photographer Amanullah Hussaini

Due to a lack of assistance, effective measures and information from the authorities as well as the many organisations, which had to leave the camp because of the lockdown, refugees from the camp showed their decision-making power and capacity to act in founding the Moria Corona Awareness Team (MCAT) in March 2020 (Fig. 1).

Omid, a young pharmacist, who has fled Afghanistan with his family, had the initial idea. Together with his friends, who are nurses, doctors and teachers from the Afghan community, they decided to raise awareness and educate people in the camp. This was first accomplished by simply walking from tent to tent and talking to the people. With this self-organised team, they wanted “to show the world and the European Union that refugees are not lazy people who only eat and sleep” but are engaged political subjects acting to improve their situation. By now MCAT is a team of 33 people who in cooperation with Moria White Helmets and the Greek organisation Stand by me Lesvos are active in health and environmental education, first aid, distribution of food and hygienic products as well as collecting waste and establishing recycling points in the camp (Omid, 2020). From 19 March, MCAT reported on their work on their Facebook page, documenting the difficult situation and problems within the camp, calling for help and evacuation (MCAT, 2020). With the help of the Danish NGO Team Humanity, who provided sewing machines and training, a tailor workshop was installed so that inhabitants could sew their own masks (Jakob, 2020).

In May 2020, MCAT together with other organisations, issued a second call demanding assistance from the EU, the governments of European countries and civil society (Moria Camp, 2020). The camp had been in lockdown since March 2020, which restricted movement to and from the camp as well as much needed health service to the camp. A medical station by Doctors Without Borders near to the camp had to close in July 2020 as doctors were threatened with prosecution by the authorities (Schmitz, 2020b).

On 2 September, the first coronavirus case was detected in Moria camp. It was a 40-year-old man from Somalia whose request for asylum had been accepted, but as he had been living in Athens on the streets, before he decided to return to the camp. The man was put under isolation while the authorities tried to trace his contacts in the camp. The whole camp was put under quarantine for 2 weeks and authorities started to test the inhabitants (InfoMigrants, 2020). Since then, tension increased due to the forced encampment under difficult conditions, the fear of becoming infected, growing mistrust among the inhabitants concerning the outbreak, as well as the measures taken. Stress, tension and unrest were rising in the camp. Within a few days, 35 people had already tested positive in the camp (Reuters, 2020). Not long before the camp burned down, there were 214 people who tested positive for the corona virus (Tagesspiegel, 2020).

### Agency under critical conditions: the fire of Moria

**Fig. 2:**
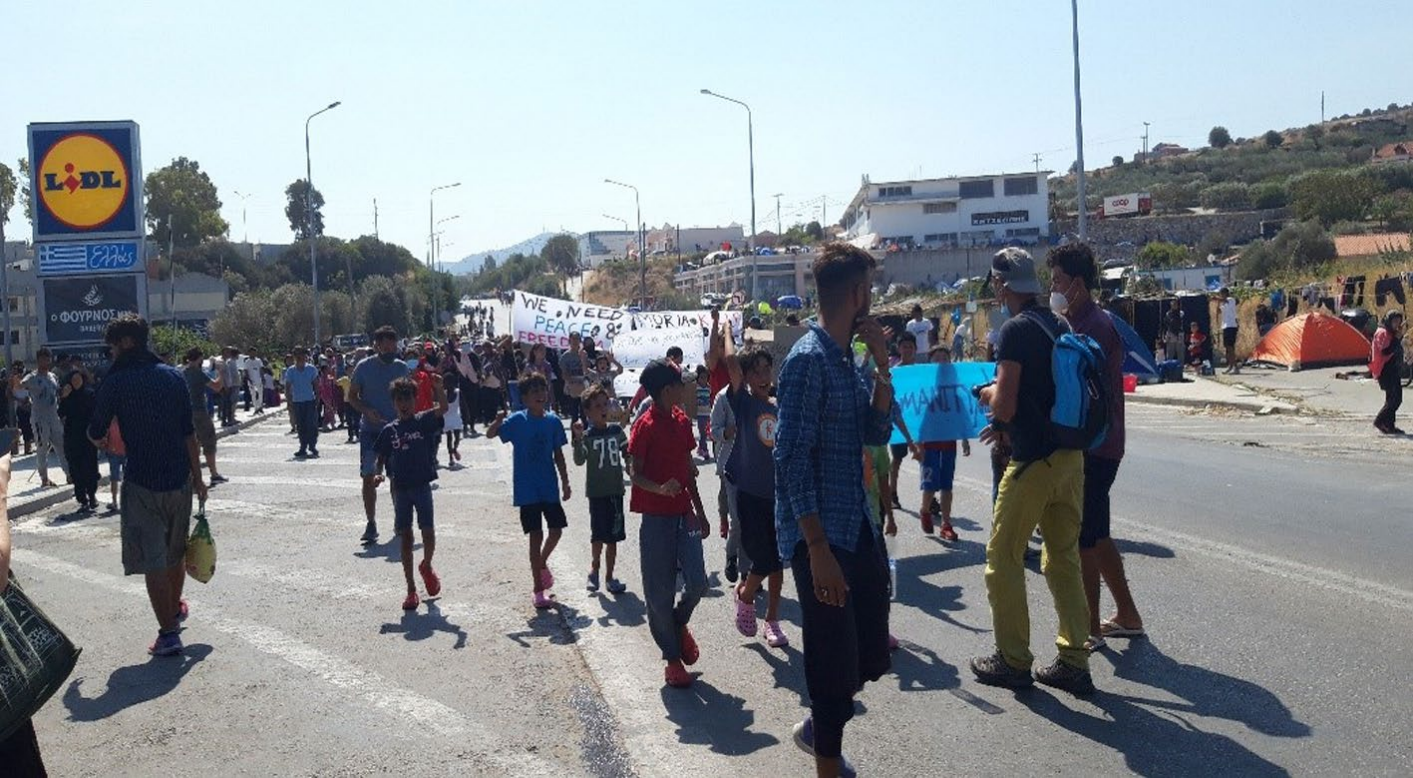
Former camp habitants of Moria protesting on the streets near Kara Tepe; Copyright and photo: Claudia Böhme 2021

On the morning of 9 September, just a few days after our arrival in Lesvos, we received news that a fire had broken out in Moria camp during the early morning hours. The fire had initially destroyed the whole administrative buildings and part of the living area, a second fire that erupted the day after destroyed the whole camp. Although it is not finally proved who laid the fire, the local inhabitants of the village, right wing activists or refugees, the police arrested six Afghan men from the camp (InfoMigrants, 2020). In March 2021, two Afghan youth were sentenced to 5 years in prison although they stated that they were not in the camp by the time of the fire (InfoMigrants, 2021). While the inhabitants fled the fire, trying to find shelter in the nearby olive groves and the city of Mytilini, Greek authorities did everything to prevent refugees from entering the city. Groups of the local population, together with right wing activists, violently hindered refugees, NGOs and us^6^ from entering Moria village and the surrounding area, seeking to prevent a reconstruction of the camp. Police roadblocks were installed on all streets leading to the camp and most refugees ended up on a main road to Mytilini. The part of the road where refugees took shelter near the already existing second camp Kara Tepe was closed by the police from both sides. When we came to the area with the help of a Greek journalist and a private helper from Athens, the former inhabitants of Moria were living next to the street in camping tents that had been provided, in self-made shelters in the bushes, under the roof of a Lidl supermarket car park, or on the unfinished roofs of warehouses next to the street. Most astonishing was how quickly people have arranged and organised themselves on the street. There was a constant movement of people going up and down the street, many carrying their remaining belongings in plastic bags or dragging food, beverages, clothes in bags, crates or dustbins behind them. Amid this chaos, they also organised themselves and showed their capacity to act in several demonstrations to demand their freedom and asylum in Europe as well as their unwillingness to return to any camp (Fig. 2).

While the refugees, aid organisations and activists demanded the evacuation of all stranded refugees to Europe, the Greek authorities were resistant as the example of the burning of a camp as a strategy to leave Lesvos could be imitated by camp inhabitants on the other islands near Turkey. The UNHCR started to build a new temporary camp near Kara Tepe but refugees actively resisted to move to the new tents, as they were afraid that this would mean another Moria for them. Finally, a delegation from the Ministry of Migration came to the area and pressed the refugees to move to the new camp if they wanted their asylum cases to be processed. Slowly, more and more people moved to the new camp but evacuation was only proceeded for a smaller part of the homeless population as most EU countries were reluctant to take refugees.^7^ Several months later, the new camp at Kara Tepe/Mavrovouni still lacked sanitation, water, drainage, health service and electricity. The situation worsened during the winter, when rains, snow and cold made life in the tents unbearable (Prange, 2021). Meanwhile, the Moria Corona Awareness Team have built up agentive structures for helping the refugees in the new camp and to report on the difficult situation.

The fire in Moria made the culmination of a crisis within a crisis as well as the failing politics resulting in the mass encampment of people under unhuman conditions visual to the public. If refugees were involved in laying the fire, this violent act can be interpreted as a form of agency and last means of resistance in this helpless situation to destroy the structures of the biopolitical power acted upon them. The peaceful and violent protests of refugees in Moria stand in line with the many example of refugees who act as political subjects who resist against unhuman forceful practices and demand for citizenship (Ataç, 2016; Kilian & Bendix, 2020; Tyler & Marciniak, 2013; Rosenberger et al., 2018).

The example of MCAT also shows a positive future oriented agency, how refugees use their resilience and agency to develop coping strategies during the coronavirus pandemic. They did not rely on help from outside but use their knowledge and competences to act for themselves.

### Coronavirus pandemic in an “accidental city”: the Kakuma refugee camp

The "Kakuma Refugee Camp" (and the later established "Kalobeyei Integrated Settlement") in Kenya, is an example of a decades-old refugee camp. Established in 1992 due to the arrival of the “Lost boys of Sudan” it has grown to a population of 196,666 registered refugees (July 2020) from over 20 countries (UNHCR, 2020b). With its diverse political, social and economic structures, the camp has according to Jansen become an "accidental city" (Jansen, 2018). Kakuma lies very marginalised in the north-western corner of Kenya, approx. 130 km to the border of South Sudan in an area with extreme climatic conditions (UNHCR Kenya, 2020). The majority of the local population are Turkana, cattle nomads, who also have difficulty accessing vital resources. This leads to violent conflicts with neighbouring groups and a tense and ambivalent relationship with the camp residents (Jansen, 2018). Life in Kakuma Refugee Camp is shaped by the hope for a life beyond the camp's borders through participation in a repatriation programme, integration into the host community or the ultimate dream, to get the chance for overseas resettlement (Böhme, 2019; Jansen, 2018; Nyaoro, 2019), a longing which is called “bufis” in Somali (Horst, 2006). However, in mid-March, all resettlement measures were stopped due to the coronavirus crisis.

**Fig. 3:**
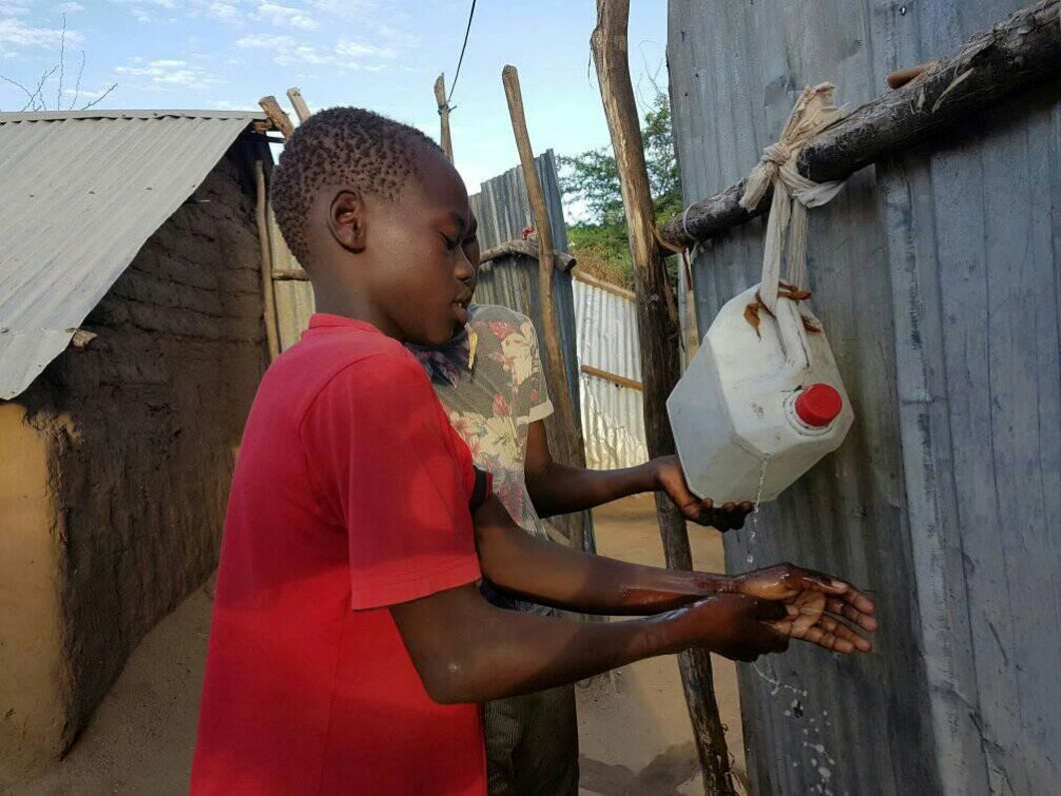
Brando Atiol, A young student (6th grade) from Jebel Marra Primary School in Kakuma Refugee Camp washes his hands on a canister that he has converted into a faucet with soap installed above it. Copyright and photo: Okello Joseph 2020

On 20 March 2020, the newspapers reported on the risk of an infected person entering the camp. The police had stopped a Somali man returning from the US in his car on the road to Kakuma who showed symptoms of the virus. He and the passengers in his car were placed in quarantine (Lutta, 2020a, 2020b). Shortly thereafter, the first rumors began to circulate that the virus had already arrived in the camp. Some communicated their worries about the health of the camp residents on Facebook along with calls for help and hygiene products. In the meantime, the inhabitants had to be creative to cope with the lack of soap and disinfectant. A post by one former resident of Kakuma on her Facebook page shows a picture of a young boy who had installed a water canister on the side of a corrugated iron hut, with soap installed above it; a water faucet for the family (Fig. 3).

At the end of March, a radio station reported that the Muslim camp residents were reciting prayers against the spread of the virus (REF FM Community Radio, 2020). On 27 March, Kenya went into Lockdown and schools, restaurants and recreational facilities had to close and a national curfew from 7 p.m. to 9 a.m. was imposed. For the camp residents this had severe effects on their well-beings as they had to remain at home in their limited dwellings. Not only did they miss education and social contacts but also economic income and the provision of food and medical care. As economic activities were slowed down, incomes severely decreased and caused lay-offs. This had negative impacts on refugee workers and their families in the camp who were not able to care for their families anymore (Boru, 2020). Moreover, the restrictions in movement caused bottlenecks in supplies for food and medicine for the camp (Rodgers, 2020). While the refugees waited for the distribution of food rations, they practiced social distancing marked out by chalk lines drawn on the ground (UNHCR, 2020a). When the first Covid-19 case was reported on May 25, the camp was officially closed for entry and exit (Nation TV, 2020). For the people living in the camp this meant they were even more imprisoned than before. But the worst effect is that durable solutions like resettlement were stopped and the hope to leave the camp for a better future shattered (Boru, 2020).

However, the camp residents organised themselves in cooperation with refugee-led organisations and social media to report on their situation. Even former camp residents called for help on Facebook and people created their own protective measures drawing on long-time agentive structures in the camp.

Dream Magical studio, a photography studio in the camp, in cooperation with other camp organisations initiated the “We are The Kakuma: Stepping up for Kakuma Refugees” crowdfunding campaign with a video showing the difficulties they faced during the crisis. With the money raised, they hired tailors, dress designers and soap makers to produce hygiene articles, and distributed hundreds of those to important professional groups and the most vulnerable in the camp.

The refugee-led independent press organ KANERE Kakuma News Reflector^8^ launched an information campaign to educate the camp residents on the virus and the risk of infection. In addition to online information about food distribution, prevention strategies, help from NGOs and confronting myths about the virus, volunteers drove around the camp with a pick-up truck and a megaphone to inform the residents in their respective languages (Boru, 2020; DW, 2020, Kanere.org).

By September 2020, more than 200 refugees in the camp had tested positive for the virus; while 142 have recovered, four people have died of the virus (A’ngela, 2020) and by the end of October the local government has declared the camp as one of the regional coronavirus hotspots (Lutta, 2020b). Objecting to reports in the media, camp inhabitants told Claudia Böhme that things were quiet and they did not even know how many people had corona in the camp.

Kakuma refugee camp stands as an example of the many forms of agency that are exhibited in a long-term huge “city-camp”. Although under vulnerable and restricted conditions, people in Kakuma display diverse forms of agency through entrepreneurial or social activity in the camp as well as their strategies to leave the camp. Moreover, the UNHCR claims to support agency and self-reliance of refugees in the camp. With traditional coping strategies developed in times of hardship as well as cultural heritage practices, it seems that inhabitants of Kakuma were well equipped to deal with the coronavirus pandemic. They quickly responded to the crisis with self-organisation and self-help using their already established knowledge, expertise, networks and resources.

## Conclusion: agency as “art of survival” in camp settings

The coronavirus pandemic has severely transformed the capacity to act for people living in refugee camps or camp-like settings around the world. Due to their characteristics and inherent structure, refugee camps have turned into places of threat and high risk of infection, isolation, violence, mistrust and fear. Firstly, the organisational structure of mass accommodation originally intended as temporary accommodation has several weaknesses, which make refugee camps hotspots for virus infection. Secondly, the institutional and system-inherent structure of camps characterised by governmentality, control and surveillance bears conflict potential, especially as refugees have already witnessed violence and trauma during their flight. The further restrictions through the application of protection measures can lead to increasing unrest, conflicts and violence in the camps. Moreover, the situation can lead to a social division of those who believe in the coronavirus pandemic and those who do not, those who comply with restrictions and those who do not, those who are or might be infected and those who are not. Culturally and socially insensitive measures and rules lead to mistrust between refugees and camp workers and administration. In the case of infection, whole refugee camps are put under quarantine for several weeks or even several months. Infected people are separated from the rest of the camp communities in isolation rooms and become outcasts.

Due to a lack of protective measures by camp administrations and governments, refugees have to take action to prevent themselves from infection. The comparative perspective shows that the different national context, histories and structures of the camps allow or hinder refugees’ agency. However, in all three cases, the different forms of agency and coping strategies of the inhabitants play a major role in coping with everyday life under the conditions of the coronavirus pandemic in the camps. As the case of German asylum homes show, protection measures were implemented from above and did not leave room for much self-initiative from the inhabitants. However, as the perception of the well organised system did not fit with mistakes and inconsistencies in the implementations of new protective measures, the inhabitants lost their trust in the camp administration. As inhabitants were more exposed to circulating rumors then to official information from the camp, mistrust and conspiracy ruled. The feeling of being imprisoned during quarantine fostered unrest and conflict in the homes. Here agency only becomes visible in mistrust, suspicion and conflict.

The cases of Moria and Kakuma show how refugees use their capacity to act and create their own protection measures through refugee led information and aid campaigns, the production and distribution of hygiene products, peaceful demonstrations and reports on their situation. If the situation becomes unbearable for camp inhabitants, most visible in Moria, their agency as political subjects demanding citizenship can also mean violent protests against their restriction of movement or against the unfair treatment and exertion of violence by securities and police. The last resort, as the example of the burning of Moria has shown, can be the active destruction of the biopolitical power structures. Our analyses showed, that agency forms and coping strategies of refugees in camps become the “habitus of the art of survival” (Seukwa, 2007: 307; 2018) during the coronavirus pandemic. These agency forms are "self-willed" practices, though which actors act according to their own values within the framework of their possibilities despite structural constraints—and situations beyond their control (see Fritsche, 2012:386). Such an understanding of agency contributes to the view that refugees are not understood as vulnerable and passive subjects. They are individuals integrated into social constellations, which results in specific possibilities and limitations of their ability to act (see also Emirbayer & Mische, 1998). This understanding of agency offers access to the dynamic processes “through which people produce ‘individual agency’ in social practices—and thus simultaneously create or implement enabling and restricting ‘social structures’” (Raithelhuber, 2012: 143). This perspective becomes more evident with the coronavirus pandemic: Refugees are dealing with the coronavirus pandemic with diverse strategies and practices, which, depending on the context, generate subjective possibilities, social positioning and status and are passed on to others as practical (functional) knowledge. Furthermore, this comparative perspective helps us to identify differently developed agency capacities of individuals, formed according to the extent of structural constraints and temporal-relational individual agency dispositions, strategies, and resources (see also Geiger, 2016).

As the the coronavirus pandemic has brought the problems and injustices of mass encampment of people to light, this crisis should be taken as an opportunity to rethink the “refugee camp” system with its common structure, rules and regulations. This would mean to actively engage in the implementation of models of alternative housing systems for refugees such as the already tested “settlement approaches” introduced in the Global South as well as decentralised forms of accommodation and solidary villages and cities in the Global North (Böhme et al., 2021).

## Data Availability

Not applicable.

## Abbreviations

EU: European Union
MCAT: Moria Corona Awareness Team
NGO: Non-Governmental Organisations
UNHCR: United Nations High Commissioner for Refugees

## Footnotes

1 Ethical Approval for fieldwork in the asylum homes was gained formally by the management as well as informally via informed oral consent from refugees taking part in the research and for the use of online postings and material gained via online communication.

2 “Trust Building and Future Construction via Smartphones and Social Media at intermediate Locations of Transnational Migration with the Example of Refugees from East Africa” funded by the German Research Foundation.

3 Name changed.

4 The number of the population of Moria are highly varying in the sources. Some estimate that in March 2020 there were 20.000 people living in the camp (Omid, 2020).

5 In spring 2020, eight EU countries had announced the transfer of 1600 especially vulnerable children to Europe. But as a result of the coronavirus pandemic this evacuation was postponed. In April 2020, only 47 children were brought to Germany, and 12 children and juveniles up to the age of 17 to Luxembourg (NDR, 2020).

6 In the night from 9 to 10 September the authors have been hindered to drive back to Mytilini and were violently attacked by a mob of people near Moria village (de Mos, 2020).

7 After heated political negotiations, Germany announced that it would bring 1553 refugees to Germany, but most of them had already been accepted for asylum in Europe (Deutsche Welle, 2020).

8 KANERE produces news, which have been published online from the camp since 2008, currently with a team of ten journalists and four editors (https://kanere.org/).
